# Exploring the Multidimensionality of Trust in Participatory Health Partnerships - A Network Approach

**DOI:** 10.3389/fpubh.2022.925402

**Published:** 2022-07-06

**Authors:** Meghan Gilfoyle, Jon Salsberg, Miriam McCarthy, Anne MacFarlane, Pádraig MacCarron

**Affiliations:** ^1^Public and Patient Involvement Research Unit, School of Medicine, University of Limerick, Limerick, Ireland; ^2^Health Research Institute (HRI), University of Limerick, Limerick, Ireland; ^3^Health Sciences Academy, University of Limerick and UL Hospitals Group, Limerick, Ireland; ^4^Mathematics Applications Consortium for Science and Industry (MACSI), Department of Mathematics and Statistics, University of Limerick, Limerick, Ireland

**Keywords:** trust, social network analysis, social networking, participatory health research, public and patient involvement (PPI), patient participation (patient engagement), community participation, community-based participatory research (CBPR)

## Abstract

**Introduction:**

Previous studies have identified “trust” as a key mechanism to achieve sustainable partnerships in participatory health research, which themselves can represent social networks. A recent review discussed the potential for social network analysis to investigate the development and maintenance of trust and its effects on partnership functioning in participatory health research partnerships. This review also recommended considering a comprehensive, nuanced and multidimensional approach to conceptualizing, operationalizing and measuring trust in research partnerships. Thus, this study aims to explore empirically the conceptualizing, operationalizing and measuring of trust in a multidimensional manner, approaching each trust dimension as an individual trust network, as well as combined as an overall trust network.

**Methods:**

We sampled the whole network, recruiting from a newly established network of 57 individuals that must collaborate to achieve a common goal. These individuals represented academic, service and community organizations of an existing participatory partnership, the *Public and Patient Involvement Ignite Network* in Ireland. Of the 57 individuals invited to take part in the study, 75% (*n* = 43) individuals completed the network survey. A survey about trust was designed based on literature in the area and was administered via Qualtrics. The survey included eight network questions: one on collaboration, and seven on specific dimensions of trust. From this, we constructed a network for each trust dimension. We compared several core network measures of each to identify structural differences between the dimensions of trust. To statistically validate them, we compared them to a random and preferential null model.

**Results:**

All the networks had a high reciprocity but were decentralized. Key differences were identified across trust dimensions, particularly in terms of integrity and shared values, visions and goals. None of the networks compared well to the null models indicating participants did not randomly or preferentially (based on how much trust they receive for a particular trust dimension) trust other partners.

**Discussion/Conclusion:**

This novel empirical social network analysis of trust in a real-world partnership elucidates the nuances and multidimensional nature of trust. This provides support for expanding this research direction to enhance understanding of and interventions for trust in participatory health research.

## Introduction

Participatory health research (PHR) has been gaining recognition on a global scale as an approach that helps to bridge the gap between knowledge and action by promoting culturally appropriate and contextually relevant research findings (1–3). Grounded in principles of social action, justice and emancipatory philosophy, PHR ensures that those who will benefit from the research findings are at the heart of the decisions making (4, 5). PHR serves as an umbrella term encompassing a variety of collaborative research approaches (i.e., community-based participatory research, integrated knowledge translation, public and patient involvement). Although these approaches may differ in origin and heritage, they all strive to bridge this gap between knowledge and practice by promoting inclusivity, while ensuring all partners for whom the research serves to benefit are actively engaged in the research process (2).

With an uptake of PHR, understanding its impact as an approach has been at the forefront for researchers in this space (1, 3, 6). Challenges remain in conceptualizing and thus articulating impact in PHR, in part due to the complex, non-linear and context-specific nature of the approach. A 2012 review by Jagosh et al. (7) highlighted several key benefits of PHR, with an emphasis on partnership synergy as a universal feature of the collaborative process necessary for building and sustaining partnerships that create resilience, sustain health-related goals, and extend program infrastructure while creating new and unexpected ideas and outcomes. Jagosh et al. (8) further explored what supports partnership synergy in successful long-term community-based participatory research partnerships. Building and maintaining *trust* was identified as a key mechanism in this process. However, Jagosh et al. (8) treated trust as a ‘black box' concept without unpacking its internal dimensions and processes.

A 2022 review by Gilfoyle et al. (9) sought to address this gap by exploring how trust is conceptualized, operationalised and measured in both PHR and social network literature. Specifically, PHR partnerships can be seen as a *social network*, defined as *connections* (i.e., *edges*) among *people* (i.e., *nodes*), organizations, or other social actors; *social network analysis* is a methodology for describing and measuring these contextual and relational dynamics among and between social actors (10). Authors from this review (9) posited that social network analysis provides tools for investigating the development and maintenance of trust and trustworthiness and their effects on partnership functioning within PHR social networks (11). Social networks have been used to explore trust in education (12), workplaces (13, 14), flood risk management (15) and even health partnerships (16, 17) but trust is not consistently and reliably conceptualized, operationalised and measured, and is often treated in an oversimplified manner. Thus, a comprehensive, nuanced and multidimensional approach to conceptualizing, operationalising and measuring trust in research partnerships is needed. When discussing the multidimensions of trust, we mean that, “the lack of consensus surrounding a definition of trust speaks to its complexity as a concept. Specifically, it is not only a psychological phenomenon but also a social one, and it can vary for each individual, across different social interactions, and across disciplines” (9).

This paper seeks to explore empirically the conceptualizing, operationalising and measuring of trust in a multidimensional manner, looking at each trust dimension as an individual trust network, and combined as an overall trust network. It is important to emphasize that in social network analysis, the networks represent the association of connections *between* individuals/organizations, not the individuals/organizations themselves.

Using an existing participatory research partnership as a case, we explore the following:

1. What are the trust characteristics at baseline of a PHR network?
2. Should trust be looked at multidimensionally?
3. Is there a relationship between the different trust networks explored?
4. Can these different networks of trust be combined to create an overall trust network? And if so, what is the relationship between the combined trust network and individual trust networks?

## Methods

This study was granted ethics approval from the University of Limerick Education and Health Sciences Research Ethics Committee (#2021_03_16_EHS).

### Setting

#### PPI Ignite Network

In 2017, the Irish Health Research Board (HRB) and Irish Research Council (IRC) funded *PPI Ignite Teams* at five universities across Ireland, to build capacity for public and patient involvement (PPI) in health research. In 2021, building on the work from the Ignite Programme, the HRB and IRC funded a *PPI Ignite Network* (2021–2026) at seven universities across Ireland, consolidating and building on the work of PPI Ignite. The PPI Ignite Network “aims to provide a shared voice for PPI across Ireland, aiming to change the research culture, and an important contributor to improving health outcomes for the public” (18).

This Network brings together academic, service and community organizations that must collaborate in an efficient, synergistic and cohesive manner to plan, implement and evaluate the PPI initiatives set out by the network. (For further information on the PPI Ignite Network see: https://ppinetwork.ie/about-us/). The Network is comprised of seven universities, a national office, 10 national-level community partners who contribute to national level governance and activities and 39 local level partners who contribute to governance and activities at one university in the Network. This participatory partnership serves as a case in which to observe the dimensions of trust in action for this study.

#### Research Advisory Group

The Research Advisory Group for this study is comprised of four research partners representing academic, service, or community organizations in the PPI Ignite Network. All members were involved in the preceding PPI Ignite grant (2017–2020) and thus have a track record of working together. This group provided input and approval for the research objectives for this social network analysis and were similarly involved with the previous scoping review (9). The group was also involved in designing the network survey, specifically by ensuring the applicability and readability of the survey. One Research Advisory Group member has been further involved in the interpretation of the results and authorship of this manuscript (co-author MM).

#### Sample

Using a sociometric (“whole network”) approach, this study aimed to recruit 57 individuals representing academic, service and community organizations acting as co-investigators and collaborating partners in the PPI Ignite Network. Each individual was invited to complete a network survey.

#### Network Survey

A network survey is a questionnaire used to generate names and connections among individuals in a network (19, 20). The network survey in this study was designed based on the dimensions of trust identified by Gilfoyle et al. (9) and in collaboration with the Research Advisory Group to ensure the appropriateness and readability of survey questions. The survey was administered electronically via Qualtrics software (Qualtrics software, Version May 2021 to August 2021). Survey questions included eight network questions: one question on collaboration from Leppin et al. (21) and seven questions that were found by Gilfoyle et al. (9) to be important dimensions of trust (see Table 1 below for a description of how each dimension was defined and measured).

**Table 1:** Descriptions of trust dimensions [based on Gilfoyle et al. (9)].

**Dimension of trust**	**Definition**	**Network question**
1 – Vulnerability	Describes the willingness of an actor (trustor) to be vulnerable to the actions of another actor (trustee). The trustor does not have complete control over how the trustee will behave and is thus, uncertain about how the individual will act, which also implies that there is something of importance to be lost, and in turn, risk involved. Therefore, to be vulnerable, there must be an opportunity for risk where the trustor must then decide if they are willing to take the risk of placing trust in the trustee. Furthermore, if there is the possibility of risk, this implies that there will be some level of uncertainty regarding how the trustee will behave. It is noted that if there is trust between partners, there is a lower level of uncertainty between how the trustee will behave. In summary, for this sub-theme we consider uncertainty and risk as necessary aspects of vulnerability.	“I would discuss with [name of network member X] how I honestly feel about my work, negative feelings and frustrations”
2 – Integrity	Concerns the extent to which the trustor thinks that the trustee will act in their best interest and the belief that the trustee will follow a set of principles, deemed acceptable by the trustor, such as they will say what is true.	“[name of network member X] keeps my interest in mind when making decisions”
3 - Reliability	Describes the confidence in and extent to which the trustor believes the trustee's will follow-through on commitments, perform a given task, and/or make decisions about something.	“[name of network member X] is dependable. For example, they stick to their word and makes sure their actions and behaviors are consistent”
4 - Ability	Describes an individual's (trustee) ability to perform a given task or make decisions about something based on their perceived skill set and competence from the perspective of another individual (trustor).	“I am comfortable asking [network member X] to take responsibility for project tasks even when I am not present to oversee what they do”
5 - Shared values, visions and goals	Highlights the need to have shared visions, values and goals in partnerships. Specifically, common goals, missions, and plans can promote trust.	“I feel that [network member X] shares a vision with PPI Ignite Networks vision and goals?”
6 - Power sharing and co-ownership	Sharing power, and fostering co-ownership in partnerships as a dimension of trust.	“I feel that [network member X] is open to discussion* about matters pertaining to the PPI Ignite Network” *Note: When we say open to discussion, we mean that this individual is willing to engage in frank, open and civil discussion (especially when disagreement exists). The person is willing to consider a variety of viewpoints and talk together (rather than at each other) and you are able to communicate with this individual in an open, trusting manner.
7 - Reciprocity	This sub-theme describes the presence of trust based on the notion that they think the trustee also trusts them back. Thus, if a trustor thinks that the trustee also trusts them, trust is thought (by the trustor) to be reciprocated (by the trustee).	“I feel that [network member X] trusts me”

Given that the networks represent the association of connections *between* individuals/organizations, not the individuals/organizations themselves, asking these questions mapped eight distinct networks. Although the overall sample consisted of the same participants, each network question mapped a distinct network about a different dimension of trust. We may then compare and contrast networks to explore, for example, if there are differences between trust networks for reliability compared with vulnerability, and so on.

To generate a network, each participant was invited to name up to seven organizations when answering the network survey questions (the same seven organizations for each question). They were asked to consider the *individual person* in the network representing these organizations when responding to the network questions. This was a noteworthy distinction as we were interested in exploring the *partnership* collaboration and trust, not trust for the organization. A list of organizations in the PPI Ignite Network was included as an attachment to the survey for reference, but participants were free to name other organizations not listed.

The first network question was a *name generator* (22), asking participants to list up to seven organizations they have collaborated with on the PPI Ignite Network. We chose this number (7) from “The social brain hypothesis” (23, 24) which estimates five as an average inner layer for core relationships. Empirical work on these layers found that they were right skewed. To account for this, we allowed participants to name up to seven organizations (not all of which have to be used) (25). The network question and scale were informed by the work from Leppin et al. (21) assessing the intensity of collaboration from [lowest level] no interaction at all, networking, cooperation, coordination, coalition, to collaboration [highest level]. Associated definitions were provided for each intensity of collaboration. Following this, participants were asked to answer seven network questions, each tapping into a dimension of trust, for the same individuals generated in the collaboration question. For example, for one dimension of trust, *vulnerability*, participants responded to the following statement: “I would discuss with [name of network member X] how I honestly feel about my work, negative feelings and frustrations.” The degree to which they related to the statement was assessed on a 5-point scale, from strongly disagree to strongly agree. The complete survey can be found in Supplementary Material 1.

### Analysis

Initially we compared the survey responses to each other calculating the correlations between the survey response for each trust item. We then constructed and analyzed the eight social networks of interest (1 re: collaboration and 7 re: trust) to obtain individual and global (or network-level) measures (or properties) described below.

#### Individual-Level Measures

*In-degree:* represents how frequently a partner was trusted on a given dimension. In-degree gives the number of edges received by a node, i.e., the number of times a person was nominated by another individual in the network (19). We also obtained the *weighted in-degree*, which represents the sum of the strengths of agreement for each trust question (described further in analysis). As discussed by Valente, 2010 (19), in-degree is one of the most useful measures for researchers as it identified opinion leaders or “popular” individuals in a network as well as being the most robust measure of centrality to missing data. This measure allowed us to identify who are the most trusted individuals for each trust dimension.^1^ We also calculated *betweenness centrality* which represents how many times a person lies on the paths between trusted partners, i.e., the frequency a node lies on the shortest path between all other pairs of nodes in the network (26). Betweenness centrality is a useful measure in this study because it identified those who occupy a strategic position in the network, acting as “gatekeepers” to those not currently connected in the network. Removing nodes with high betweenness can lead to the network becoming disconnected, i.e., breaking the structure of the network down into more than one component (27).

#### Network-Level Measures

*Average In-degree*: looks at the mean number of received nominations across the network. This helped us identify how high trust is overall in the network. *Clustering coefficient:* measures the degree to which there are dense pockets of interconnectivity in the network (i.e., clumpiness) (19). Thus, a high clustering coefficient means if you trust two people, they are also likely to trust each other. Measuring the clustering coefficient helped us to identify if there were certain trusting groups throughout the network. *Assortativity:* measures the tendency for nodes to connect to nodes that are similar to themselves (28). It is related to the notion of homophily (that nodes link to those similar to themselves). For example, assortativity is positive if people with a high in-degree have a higher tendency to connect to nodes who also have a high in-degree. Assortativity is negative if those with a high in-degree are more likely to connect with others of a low in-degree. *Reciprocity:* occurs when edges go both ways. For example, if both individuals agreed or strongly disagreed with the same trust dimension, then reciprocity was present. This was important to measure as reciprocity is described as an important mechanism of trust (29, 30). *Freeman Centralization about the In-degree:* measures whether the network is centered around a small group of individuals, i.e., the degree to which the edges of a network focus around an individual or a set of individuals (19). If the network was centralized, it meant that one or a few individuals were in a position of power and control; decentralized would imply the opposite, where the power and control were distributed across many individuals. These measures allowed us to compare the structural properties of trust dimensions.

#### Pearson Correlations

Before constructing the networks, we calculated Pearson correlations between each pair of survey questions where a correlation of one implied each entity answered the same response value to each question for everyone named. This gives an indication as to how similar the response to the individual trust items may be before taking a more fine-grained network approach.

#### Individual Trusts Networks

We constructed individual trust networks derived from each of the seven dimensions of trust explored (i.e., seven trust dimension questions) in the survey. This was done by assigning a value from −1 to +1 depending on the selection of strongly disagree to strongly agree (in intervals of 0.5 for the 5-point scale) for each network question. Specifically, when a participant responded ‘agree', an edge weight of 0.5 was added, while “strongly agree” added an edge weight of one. An edge was not added if participants responded with “neither agree nor disagree,” “disagree,” or “strongly disagree” identifying only a presence of, or absence of, a trust edge. This is because, in alignment with the literature, we did not want to infer neutral agreement or disagreement with each statement as an expression of distrust. Specifically, distrust differs conceptually from trust (31) and more specifically stated by Jones (32), “the absence of trust is not to be equated with distrust.”

From these seven trust dimensions, we created an 8th trust network we referred to as *combined trust*. For combined trust, we took all the edges from each of the seven trust dimension questions and assigned an average weight. Thus, if a participant strongly agreed with each question on the network survey, they were present in the combined trust network with a weight of one. If, for example, they strongly agreed with one question and disagreed with the rest, they had a weight of 1/7.

#### Spearman Correlations

On an individual level, we tested whether the nodes with the highest weighted in-degree and betweenness centrality were consistent in each network. We did this by ordering the nodes, from lowest to highest quantities (for weighted in-degree and betweenness centrality scores), and then performing a Spearman correlation on the rank. To maintain an increasing rank, distinct values were required (i.e., we did not include many nodes at degree 0 as they could not be ranked in a meaningful order), so we limited the correlation to the top 20 nodes in each measure. We only reported correlations that were significant below *p* < 0.05.

Finally, to statistically validate these results, we proposed two null hypotheses. The first randomly selected the number of neighbors for each node as well as randomized the value for their survey scores. This random null model would represent the case where participants randomly filled out the survey. From these, we created the networks as described above and compared the results to the random model.

The second simulation generated networks of the same size using the *preferential attachment* model (33). This model is designed to emulate many real-world complex networks where nodes aim to connect to popular nodes (i.e., high incoming connections for that trust dimension). From this, we identified if nodes are preferentially connected to nodes with a high degree. This yielded a complex network with a high clustering coefficient and Freeman centralization allowing us to statistically compare the values from the trust networks and identify whether people are connecting to organizations with high trust preferentially or if some other mechanism is responsible for the structure of the network.

For both models, a simulation ran 1,000 iterations measuring the same network quantities described above. From these simulations, the 2.5 and 97.5 quantiles for each network score were taken. If the value of the data was outside this range, we said the 95% confidence interval is outside the random or preferential model.

## Results

Of the 57 individuals invited to take part in the study, 75% (*n* = 43) individuals completed the network survey. This included 100% (*n* = 8) of the site leads and the national office, 80% (*n* = 8) of the national partners, and 69% (*n* = 27) of local partners involved in the study.

As shown in Table 2, findings indicated a statistically significant positive correlation across all trust dimensions (*p* < 0.001), but the positive correlations varied in the strength of correlation. For example, responses for trust dimensions 2 (integrity) and 3 (reliability) were the most highly correlated (*r* = 0.70), while trust dimensions 1 (vulnerability) and 6 (power sharing and co-ownership) were the most different (*r* = 0.4) these findings suggested that individuals who deem others to be reliable, often also thought they had integrity. Comparatively, if others agreed or strongly agreed that they would be vulnerable to a named individual, they were *less likely* to respond similarly to power sharing and co-ownership with that same-named other.

**Table 2:** Pearson correlations for trust networks.

**Networks** **(*n* = 59)**	Combined Trust*	**Trust network 1^**a**^** **(Vulnerability)**	**Trust network 2^**b**^** **(Integrity)**	**Trust network 3^**c**^** **(Reliability)**	**Trust network 4^**d**^** **(Ability)**	**Trust network 5^**e**^** **(Shared values, visions and goals)**	**Trust network 6^**f**^** **(Power sharing and co-ownership)**	**Trust network 7^**g**^** **(Reciprocity)**
Combined*trust								
**Trust dimension 1**^**a**^ (Vulnerability)	0.79							
**Trust dimension 2**^**b**^ (Integrity)	0.87	0.67						
**Trust dimension 3**^**c**^ (Reliability)	0.85	0.59	0.7					
**Trust dimension 4**^**d**^ (Ability)	0.82	0.58	0.69	0.64				
**Trust dimension 5**^**e**^ (Shared values, visions and goals)	0.73	0.55	0.59	0.57	0.47			
**Trust dimension 6**^**f**^ (Power sharing and co-ownership)	0.73	0.4	0.56	0.66	0.44	0.66		
**Trust dimension 7**^**g**^ (Reciprocity)	0.82	0.56	0.64	0.68	0.67	0.53	0.6	

^*^Sum of scores of trust questions divided by the number of trust questions (7) ^**a**^Trust Network 1 question “I would discuss with [name of network member X] how I honestly feel about my work, negative feelings and frustrations,” ^**b**^Trust Network 2 question “[name of network member X] keeps my interest in mind when making decisions”, ^**c**^Trust Network question: “[name of network member X] is dependable. For example, they stick to their word and makes sure their actions and behaviours are consistent; ^**d**^Trust Network 4 question: “I am comfortable asking [network member X] to take responsibility for project tasks even when I am not present to oversee what they do,” ^**e**^Trust Network 5 question: “I feel that [network member X] shares a vision with PPI Ignite Networks vision and goals?”, ^**f**^Trust Network 6 question: “I feel that [network member X] is open to discussion^*^ about matters pertaining to the PPI Ignite Network,” ^**g**^Trust Network 7 question: “I feel that [network member X] trusts me”.

**Figure 1:**
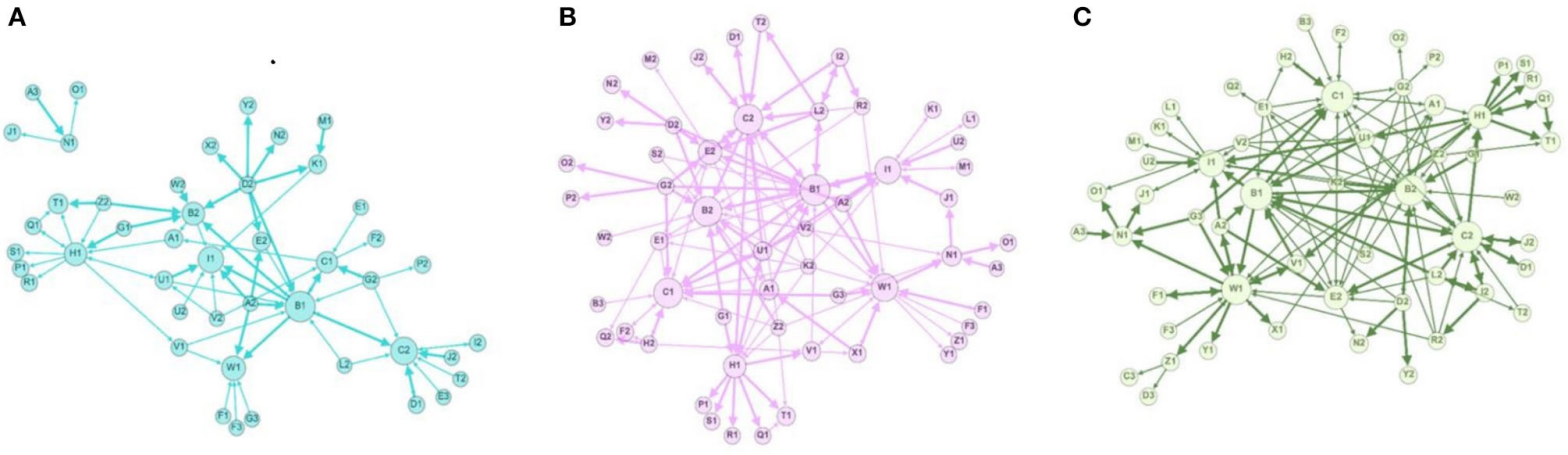
Networks for trust dimensions 1 (vulnerability), 5 (shared values, visions and goals) and 6 (power sharing and co-ownership). **(A)** Network for trust dimension 1-vulnerability. This network was mapped by asking individuals to answer a question pertaining to vulnerability, specifically: “I would discuss with [name of network member X] how I honestly feel about my work, negative feelings and frustrations”. **(B)** Network for trust dimension 5-shared values, visions and goals. This network was mapped by asking individuals to answer a question pertaining to shared values, visions and goals, specifically: “I feel that [network member X] shares a vision with PPI ignite networks vision and goals?”. **(C)** Network for trust dimension 6-power sharing and co-ownership. This network was mapped by asking individuals to answer a question pertaining to power sharing and co-ownership, specifically: “I feel that [network member X] is open to discussion* about matters pertaining to the PPI ingnite network”.

These nuances between trust dimensions were further explicated when exploring network measures for each one (i.e., weighted in-degree, number of edges) (shown in Table 3 below). Like the findings discussed in Table 2, we saw the largest contrast between the networks for trust dimension 1 (vulnerability) and trust dimension 5 (shared values, visions and goals) and 6 (power sharing and co-ownership), but also trust dimension 2 (integrity) to trust dimension 5 (shared values, visions and goals) and 6 (power sharing and co-ownership). For example, the number of edges for networks mapping trust dimensions 1 (vulnerability) and 2 (integrity) was nearly half that of trust dimensions 5 (shared values, visions and goals) and 6 (power sharing and co-ownership). This implied that people agreed or strongly agreed to statements about shared values, visions and goals as well as power sharing and co-ownership, but were much less likely to agree or strongly agree with statements about vulnerability and integrity. Trust dimensions 1 (vulnerability) and 2 (integrity) also had a lower weighted in-degree and were less likely to have reciprocal edges compared to trust dimensions 5 and 6. We further highlighted some of these findings in Figures 1A–C below, where we mapped three networks for trust dimensions 1, 5 and 6. These networks were chosen to visually demonstrate some notable structural differences at both the individual and network levels. At the individual level, node size was proportional to the weighted in-degree. Furthermore, when looking at Figure 1A. Network for Trust Dimension 1 – *vulnerability*, a cluster of four nodes appears to be disconnected from the network. This may be because although the study partnerships consist of lead sites that were part of the initial grant (2017–2020), as well as lead sites new to the second grant (2021–2026), all had the opportunity to bring in local partners that may not have existed in the first round. Thus, at the time of this survey, some were new to the network and had not yet had the opportunity to interact with other members of the partnership, although they may have interacted with each other. Therefore, they may appear in network maps (i.e., vulnerability) as isolated clusters.

**Table 3:** Social network analysis across trust networks.

**Networks (*N* = 59)**	**Number of edges**	**Weighted in-degree mean (std)**	**Clustering coefficient**	**Weighted assortativity**	**Weighted In-degree centralization**	**Reciprocity**
**Combined trust**	136	3.13 (4.45)	0.25	−0.31	0.32	0.46
**Trust dimension 1**^**a**^ (Vulnerability)	73	1.70 (2.96)	0.07	−0.16	0.18	0.29
**Trust dimension 2**^**b**^ (Integrity)	73	1.56 (2.70)	0.07	−0.24	0.19	0.36
**Trust dimension 3**^**c**^ (Reliability)	118	3.14 (4.35)	0.12	−0.24	0.27	0.38
**Trust dimension 4**^**d**^ (Ability)	90	2.20 (3.73)	0.04	−0.25	0.21	0.31
**Trust dimension 5**^**e**^ (Shared values, visions and goals)	145	3.64 (5.60)	0.17	−0.28	0.33	0.48
**Trust dimension 6**^**f**^ (Power sharing and co-ownership)	142	3.41 (5.01)	0.14	−0.3	0.28	0.45
**Trust dimension 7**^**g**^ (Reciprocity)	109	2.41 (3.74)	0.11	−0.24	0.23	0.44
**Collaboration**	137	7.814 (11.173)	0.20	−0.26	0.66	0.45

^**a**^Trust Network 1 question “I would discuss with [name of network member X] how I honestly feel about my work, negative feelings and frustrations,” ^**b**^Trust Network 2 question “[name of network member X] keeps my interest in mind when making decisions”, ^**c**^Trust Network question: “[name of network member X] is dependable. For example, they stick to their word and makes sure their actions and behaviours are consistent; ^**d**^Trust Network 4 question: “I am comfortable asking [network member X] to take responsibility for project tasks even when I am not present to oversee what they do,” ^**e**^Trust Network 5 question: “I feel that [network member X] shares a vision with PPI Ignite Networks vision and goals?”, ^**f**^Trust Network 6 question: “I feel that [network member X] is open to discussion^*^ about matters pertaining to the PPI Ignite Network”, ^**g**^Trust Network 7 question: “I feel that [network member X] trusts me”.

On across-network *similarities*, we noted that the trust dimension networks were disassortative, indicating that the nodes with high in-degree were less commonly linked to those with high in-degree compared to nodes with a lower in-degree. Therefore, those who received a lot of incoming edges for a particular trust dimension network were not likely to connect to others who received a lot of incoming edges for that same trust dimension network. This contrasts with existing literature, as social networks tend to have positive values of assortativity where people often associate with those similar to themselves (34). Further, we saw that all trust dimension networks were relatively decentralized, indicating that edges were generally dispersed across nodes in the network.

**Figure 2:**
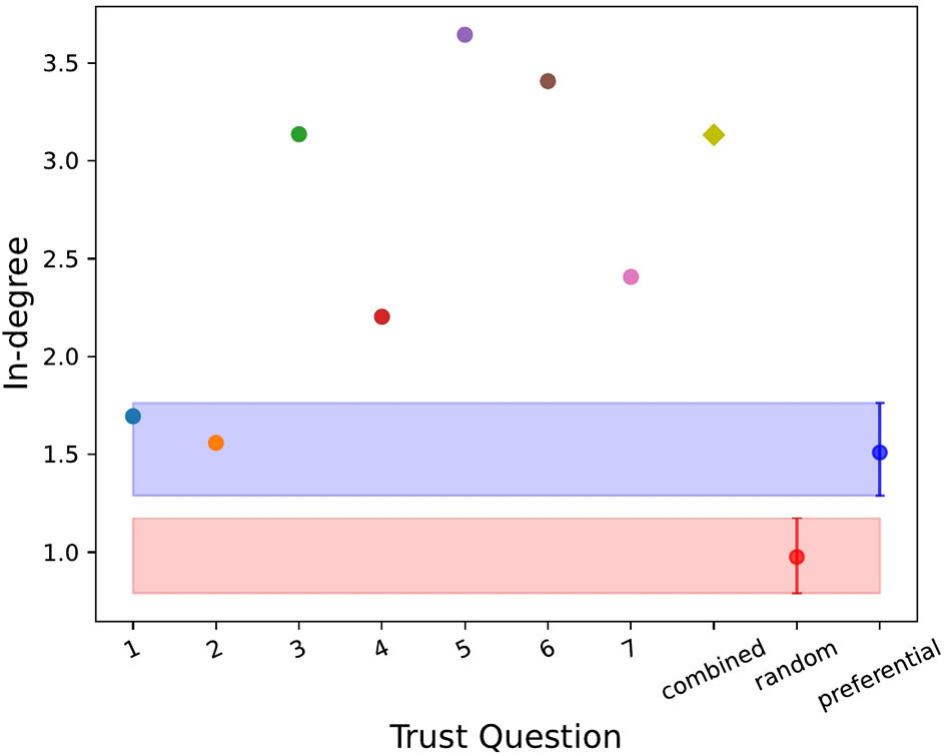
The weighted in-degree for each trust question (i.e., dimension of trust), the combined trust network (yellow diamond) and the random (in red with the mean and 2.5 and 97.5 quantiles around it) and preferential (in blue with the mean and 2.5 and 97.5 quantiles around it) null models. Trust network 1 (vulnerability) and 2 (integrity) showed similar in-degree behavior to the preferential model, however the random model did not perform well as expected.

**Figure 3:**
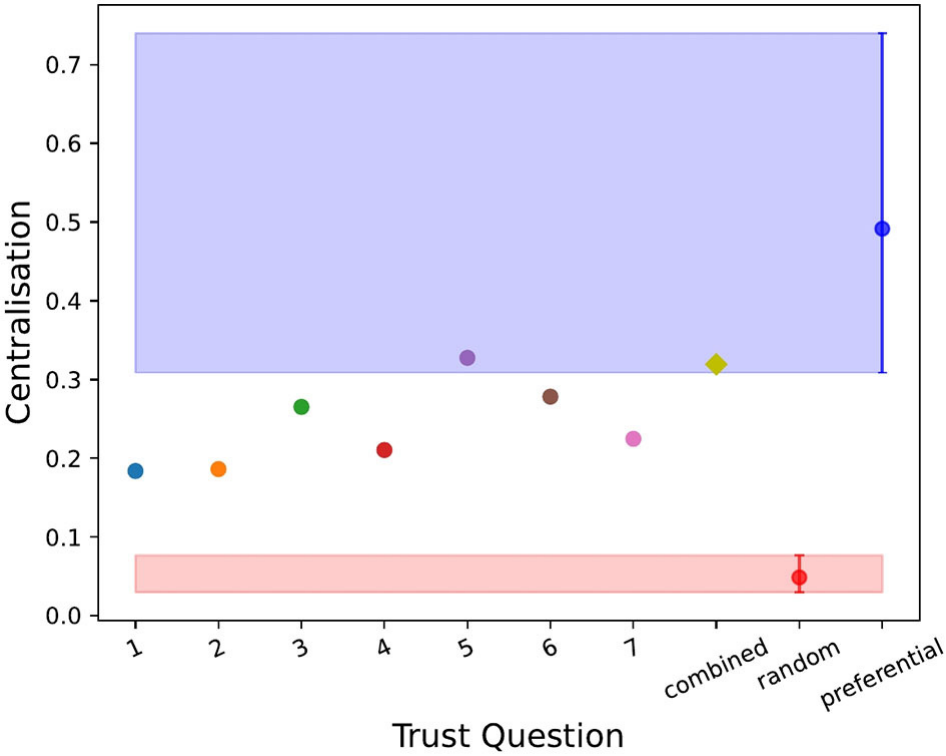
The weighted Freeman centralization about the in-degree for each trust question (i.e., dimension of trust), the combined trust network (yellow diamond) and the random (in red with the mean and 2.5 and 97.5 quantiles around it) and preferential (in blue with the mean and 2.5 and 97.5 quantiles around it) null models. Trust questions 5 and the combined network showed similar behavior to the preferential model, however, the random model yielded low values of centralization.

**Figure 4:**
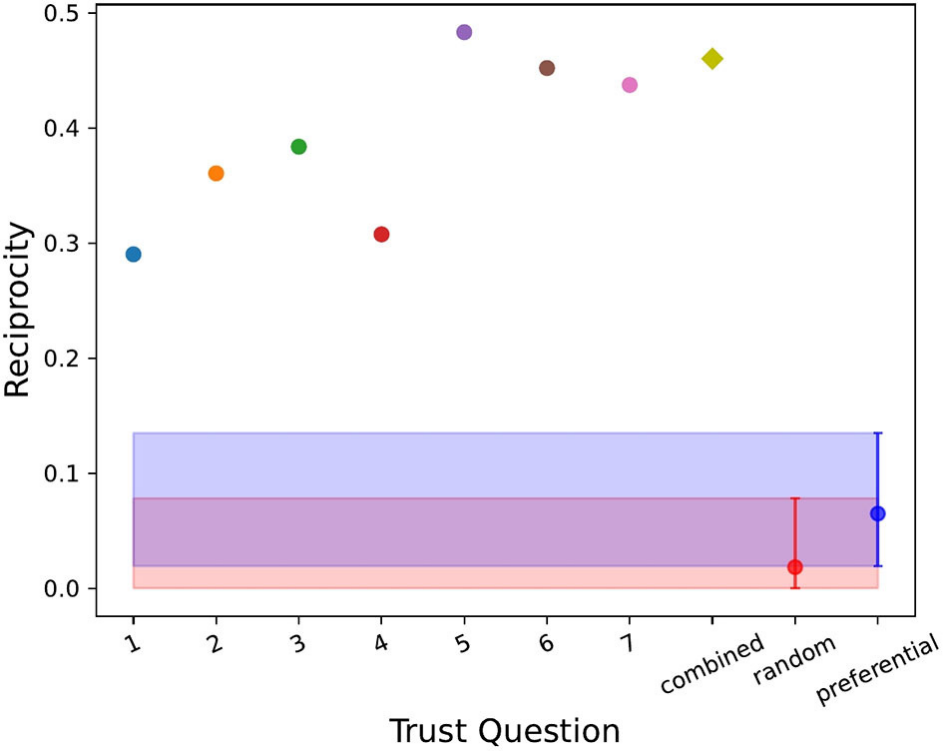
The reciprocity for each trust question (i.e., dimension of trust), the combined trust network (yellow diamond) and the random (in red with the mean and 2.5 and 97.5 quantiles around it) and preferential (in blue with the mean and 2.5 and 97.5 quantiles around it) null models. All the networks were beyond the 95% confidence interval for each null model.

To statistically validate the networks quantities in Table 3, we compared the results to the two null models described above, random and preferential. Figures 2–4 show the values of the weighted in-degree, Freeman centralization on the in-degree and the reciprocity for each network as well as the two null models with the 2.5 and 97.5 quantiles around their means from the simulations. Neither model performed well in these measures for each network. The range in assortativity (found in Supplementary Material 2) was very large in the null models and all networks fell within the 95% confidence interval. The clustering coefficient (found in Supplementary Material 2) for each network was outside the 95% confidence interval for the random model, but within for the preferential model. Comparatively, for the other three measures, the actual values were rarely in the 95% confidence interval for either null model, implying that overall, none of the networks were well described by the null models.

From this, we concluded that for each of the trust networks, neither the random nor preferential model successfully explained the data. Therefore, trust relied on some other mechanism for the formation of these networks. We also observed that the Freeman centralization scores here were low when compared to the preferential model, indicating that these networks were decentralized apart from trust dimension 5 (shared values, visions and goals). Similarly, all of these networks had high reciprocity relative to the two null models and thus reflect specific/authentic characteristics of the PPI Ignite Network under analysis.

Also in Table 3, we presented results for the collaboration network. This network utilized a different scale to that of the trust network questions, assessing the level of collaboration. On this scale each response connected the nodes with each increasing value implying further strength of collaboration. Each edge represented a score (i.e., weight) from 1–5 based on survey responses for collaboration [a similar process to the trust network scores described above (see *Individual trusts networks* in Analysis)]. This led to a higher weighted mean in-degree (7.814). Furthermore, the network had different properties compared to the others, such as a higher centralization (0.66).

### Spearman Correlations

The following trust questions were found to be correlated by weighted in-degree: combined trust network and trust dimension 1 (i.e., network) (vulnerability) (*r* = 0.45, *p* = 0.04), trust dimensions 2 (integrity) and 3 (reliability) (*r* = 0.46, *p* = 0.04), trust dimensions 5 (shared values, visions and goals) and 7 (reciprocity) (*r* = 0.52, *p* = 0.02), trust dimensions 6 (power sharing and co-ownership) and 7 (reciprocity) (*r* =0.56, *p* = 0.01) and the strongest, trust dimensions 5 (shared values, visions and goals) and 6 (power sharing and co-ownership) (*r* = 0.91, *p* < 0.01).

For betweenness centrality, the following trust questions were found to be statistically significantly correlated (*p* < 0.05): trust dimensions 5 (shared values, visions and goals) and 6 (power sharing and co-ownership) (*r* = 0.47, *p* = 0.03), trust dimensions 4 (ability) and 6 (power sharing and co-ownership) (*r* = 0.51, *p* = 0.02), trust dimensions 2 (integrity) and 4 (ability) (*r* = 0.55, *p* = 0.01), trust dimensions 3 (reliability) and 4 (ability) (*r* = 0.56, p=0.01), trust dimensions 3 (reliability) and 6 (power sharing and co-ownership)) (*r* = 0.68, *p* < 0.01). This implied that the highest-ranking individual nodes for each of these networks were very similar to one another.

### Summary

In summary, when exploring trust in a multidimensional way using social network analysis, we identified key baseline trust characteristics of the PPI Ignite Network **(RQ#1)** based on 43 completed network surveys. Specifically, we found that the trust networks were relatively decentralized overall, indicating that the trust connections were not, from a network level, focused on a cluster of key individuals. Furthermore, the trust edges had a high degree of reciprocity, indicating that the trust edge often went both ways. As discussed previously, this is important as reciprocity has been discussed as an important mechanism of trust (29, 30). We also found a high mean weighted in-degree across the trust networks. This indicated that the same individuals have the highest number of incoming trust edges across the trust networks. However, the number of incoming trust edges differed depending on the trust network explored. Thus, although the same individuals received the highest number of incoming edges across the different trust networks, the *number* of edges differed. For example, the highest mean weighted in-degree for the integrity (trust dimension 2) was 1.56 trust edges, while for shared values, visions and goals (trust dimension 5), the mean weighted in-degree was 3.64.

This revealed some of the nuances and complexities of trust when looking at trust multidimensionally and from a network perspective **(RQ#2)**. From the baseline characteristics explored, we noted some similarities across the different trust dimension networks (i.e., overall centralization), but also important network differences that may not have been revealed if looked at in a unidimensional or binary way (i.e., who do you trust?).

When further exploring the relationship between the different trust dimensions (i.e., networks) **(RQ#3)**, we found that they were positively correlated with each other at a statistically significant level but varied in terms of the strength of correlation i.e., trust dimensions 2 (integrity) and 3 (reliability) [*r* = 0.7] and trust dimensions 5 (shared values, visions and goals) and 6 (power sharing and co-ownership) [*r* = 0.66]. This indicated that certain trust dimensions were more alike than others.

Finally, we found that when exploring trust in combination (i.e., all trust dimension networks combined into one overall trust network compared to individual trust networks) **(RQ#4)** the trust network with the largest network measures (i.e., reciprocity) tended to dominate the network properties of the combined trust network. This suppressed important differences that were found at the individual level. For example, as shown in Figure 3, the combined trust network appeared to be centralized compared to the preferential model, while six of the seven individual networks demonstrated decentralization. This indicated that networks with lower values were suppressed by the combined trust network. Thus, like the findings discussed for RQ#2, when we combined trust networks into one overall trust network, important nuances may have been lost.

## Discussion

This paper provides empirical support for the findings discussed in the review by Gilfoyle et al. (9). We will explicate this support and how it compares with the wider literature for each research question below.

### Research Question 1

The trust networks are *not* dominated by a few central individuals and are relatively dispersed for each of the trust networks. This may be surprising as the PPI Ignite Network was set up with a central administrative structure, mirroring a hub and spokes model, indicating the potential for an inherently centralized structure. However, in the setup of the PPI Ignite Network, resources and decision-making pertaining to goals and objectives were distributed across the Network. In other words, the partnership was set up to be an administratively centralized network, but a power distributed network. This is very similar to, for example, the way universities are set up, with a very hierarchical administrative structure, yet academic resources and decision-making distributed among departments and individual faculty-members. This meant that collaboration and opportunities for trust were dispersed throughout the PPI Ignite Network. Therefore, this analysis provides important empirical evidence about the value of the Network's set up. This contrasts with a *collaboration* network explored in the study by McMullough et al. (16) who found the network (*n* = 41) to be highly centralized. This, however, could be because their survey was administered at a point in time when the network had been collaborating for several years, thus the partnerships could have been well established and strengthened. Similarly, Barnes et al. (35) found both a high degree centralization for both collaborative ties and trust ties in their network of swimming providers (i.e., lessons and/or programs) comprised of 25 individuals representing 25 organizations. However, both studies (16, 35) discussed both benefits and challenges for the network with a high degree centralization. Specifically, in that it helps with efficiency of the network *if* the central individuals are “positive” leaders, but can also create bottlenecks, and reduce the dissemination of information as information must flow through these central individuals before reaching others in the network (16, 35).

### Research Question 2

Landmark studies of participatory health research, such as Jagosh et al. (7) identify trust as a critical mechanism underlying partnership function. However, their treatment of trust as a “black box” concept makes it difficult to measure or address, in order to improve partnership outcomes. It is beneficial to conceptualize, operationalise and measure trust multidimensionally to ensure a comprehensive understanding of how trust is operating in a partnership. Specifically, this analysis shows the ways in which certain dimensions of trust, may be more prominent in a network (i.e., shared values, visions and goals), compared to others (i.e., integrity). Thus, the way to strengthen dimensions of trust in a network, such as through structural interventions (i.e., strategic actions that or remove links between nodes) (36), should differ depending on which trust dimension is/is not prominent in a Network. This finding is an especially important contribution to the literature as it is the first study, to our knowledge, exploring empirically the multidimensionality of trust using social network tools, by comprehensively mapping individual trust dimensions. For example, a study by Gursakal et al. (13) investigated general trust pertaining to the entire network by tapping into three trust dimensions (ability, benevolence and integrity), but mapped trust networks more broadly by asking trust in a binary manner: “who do you trust and in which level.” Consequently, structural interventions could not be recommended based on different trust dimensions as per the findings we reported here.

### Research Question 3

There is a relationship between the trust dimension networks explored, but some are more correlated than others. For instance, power sharing and co-ownership was strongly correlated with shared values, visions and goals and reliability, but only weak to moderately correlated with vulnerability and ability, respectively. Meanwhile, ability and reliability are strongly correlated with each other. Of the studies retained in the scoping review by Gilfoyle et al. (9) that used multiple dimensions - and therefore multiple network questions - to investigate trust, none explored the correlation between these trust dimensions. For instance, Ardoin et al. (12) investigated multiple trust dimensions and network questions pertaining to reciprocity, vulnerability, dependability, and reliability, but do not appear to explore if these dimensions are correlated. It is also unclear if they combined these trust dimensions into an overall trust network or explored trust dimensions as individual trust networks. Similarly, as described above, Gursakal et al. (13) also did not examine if trust dimensions are correlated with each other. Thus, our findings add to the literature by elucidating such correlations.

### Research Question 4

Although individual trust dimensions were combined to create an overall trust network, like with RQ#2, important nuances were lost when combined as one overall trust network, compared to when the trust dimensions were looked at individually. For example, when combined, the network might appear to be more centralized overall. Further, as discussed in RQ#2, we would not be able to identify important individual trust dimensions differences (i.e., integrity dimension vs. shared values vision and goals dimension). For instance, Zhou et al. (37) combined responses to three trust dimensions (ability, reliability, and friendship) to create one weighted trust score, limiting the ability to explore specific nuances of these dimensions, such as is one stronger in the network than another? And if so, how does this impact the network? Zhou et al. (37) further highlight the subjectiveness of trust as a concept, and the need to “design more comprehensive ways for quantifying the relationship.” Although this is seemingly in reference to other important relationship networks (beyond exploring trust, communication and supervision), it can also be applied to the measurement of trust.

### Strengths and Limitations

This study has several noteworthy strengths, addressing current gaps in the trust, social network and participatory health research literature. First, we consistently conceptualized, operationalized and measured trust in a comprehensive way, drawing on the unique experiential expertise of the Research Advisory Board to ensure context appropriateness. This is especially useful, as trust depends on context (38), and involving Research Advisory Board members in the survey development and design who are also involved in the network, helped ensured relevancy and feasibility of the survey, important for our study context. Secondly, this study utilizes an interdisciplinary approach to measuring trust, by incorporating principles and techniques of network science and social science. As discussed by Lewicki and Brinsfield (39), trust is an interest across disciplines, but is often explored within a single discipline. Indeed, convergence across disciplines in how we conceptualize, operationalize and measure trust is important, and as illustrated in this study, can reveal unique insights and solutions often not considered. As highlighted by Lucero et al. (40), “by better understanding trust, we can better understand its process.” Furthermore, this study attests to the feasibility of generating and employing a network survey that operationalizes trust in a multidimensional way, which is not overly burdensome on participants. As discussed by Ferrin et al. (41), we also recognize the challenges of exploring trust so comprehensively in a network analysis setting. However, we feel this can be mitigated by seeking stakeholder input and streamlining the process for survey administration. Specifically, involving a Research Advisory Board can then help guide feasible and context-appropriate networks surveys and questions (i.e., limiting the number of names to input for each question), and availing of web mechanics offered in survey software (i.e., auto-population of fields as much as possible based on previous question selection) helps to reduce the information participants need to manually input into the survey.

Study limitations should also be considered. First, our study was cross-sectional. This is limiting as trust is a dynamic construct that is always changing (40, 42) and should be measured over time. We are currently planning for a follow-up study to explore trust at more than one time point. Secondly, from discussions with our Research Advisory Board, we thought it appropriate to measure the strength and quality of a relationship, a dimension of trust revealed by Gilfoyle et al. (9), by assessing the strength and level of collaboration as opposed to asking questions about friendship, which may not be relevant to this type of network. However, as collaboration was asked on a different scale to other trust dimensions, it was difficult to assess its correlation to other trust dimensions. Therefore, we decided to exclude it in the correlation measurements. Finally, network findings on their own can be limited to the interpretation of the researcher and the network questions posed, underscoring the importance of employing mixed methods when interpreting the network results (i.e., follow-up interviews with certain individuals in the network to verify findings). Although we employed strictly quantitative methods in this study, the consultation with the Research Advisory Board, one of which was involved in the interpretation of the network findings (co-author MMC), provides contextual support for the findings.

### Implications for Research and Future Directions

This research provides empirical evidence in support of findings revealed by Gilfoyle et al. (9) and explicates several key considerations for researchers. First, we understand that the relational dimensions of trust are inherently complex, and depending on the context, may not always be relevant or appropriate when creating a network survey. Therefore, we encourage researchers at minimum, to consult with those who hold unique experiential expertise of a network when deciding which trust dimensions are most appropriate for their research context. As trust is seen as a key mechanism in partnership functioning, those interested in understanding how and why partnerships succeed or fail need to carefully match the aspect of partnership function they are examining to the correct trust dimension. This has implications for fully understanding the several conceptual models that have been proposed for participatory research. We also urge researchers to consider an interdisciplinary lens when tackling complex conceptual and operational issues about trust (and other relational constructs), that both fall within and extend beyond their discipline, and to move away from reducing trust to a binary form (present/absent). Finally, it is important to consider trust dimensions as individual networks to ensure a nuanced understanding of trust in a network. This is helpful for identifying and applying appropriate structural interventions to enhance trust in a specific network, and ultimately the likelihood of successful outcomes of the partnership.

We understand trust changes over time, and not exclusively in a linear manner (40) (i.e., lack of trust to trust). Thus, we plan to conduct a follow-up study exploring trust longitudinally and employing a mixed-methods study design, adding to the robustness of these findings. Specifically, we explore if these methodological techniques reveal insights into how trust changes over time in a network/partnership, if this varies depending on length of time in the network/partnership, and if certain combinations of trust dimensions could be grouped together. Finally, although non-systematic consultation was appropriate for our purposes here, future research could investigate more generally if and what dimensions of trust are important for different types of partnerships and collaboration. For example, although the trust dimension for vulnerability had fewer connections than that of power sharing and co-ownership, it may not be as important in certain contexts.

## Conclusion

In conclusion, this study provides empirical evidence that there is merit in investigating trust both consistently (i.e., measured in line with how it is defined and operationalized) and in a multidimensional manner. As the first study to our knowledge examining trust in this way, we hope this work provides empirical and conceptual clarity for exploring trust in partnerships and encourages future research that will add to these findings.

## Data Availability Statement

The raw data supporting the conclusions of this article will be made available by the authors, without undue reservation.

## Ethics Statement

The studies involving human participants were reviewed and approved by University of Limerick Education and Health Sciences Research Ethics Committee (#2021_03_16_EHS). The patients/participants provided their written informed consent to participate in this study.

## Author Contributions

MG conceptualized and led the study, drafted, and edited the final manuscript. PMC secured funding, analyzed the data and contributed to the study design, data analysis and interpretation, writing, and editing of the manuscript. JS secured funding, contributed to the study conceptualization, data analysis and interpretation, and contributed to the writing and editing of the final manuscript. AM and MM contributed to study conceptualization, interpretation of results, and reviewed and approved manuscript. All authors have made substantive intellectual contributions to the development of this study.

## Funding

MG was supported by the GEMS-10 scholarship from the University of Limerick (Ireland), and a scholarship from the Integrated Knowledge Translation Research Network (Canada: CIHR Foundation Grant; FDN #143237). PMC was funded by Science Foundation Ireland, grant number 16/IA/4470.

## Conflict of Interest

The authors declare that the research was conducted in the absence of any commercial or financial relationships that could be construed as a potential conflict of interest.

## Publisher's Note

All claims expressed in this article are solely those of the authors and do not necessarily represent those of their affiliated organizations, or those of the publisher, the editors and the reviewers. Any product that may be evaluated in this article, or claim that may be made by its manufacturer, is not guaranteed or endorsed by the publisher.

## Footnotes

^1^We do not present out-degree as each organisation had a limit to how many organisations it could name (i.e., there is a maximum out-degree of 7). Also, not every organisation named was surveyed. Therefore, we only used the out-degree to calculate the reciprocity including in this calculation only individuals that were surveyed.
