# Supplementary figures and images for: Exploring the Multidimensionality of Trust in Participatory Health Partnerships - A Network Approach
Source: Front Public Health. 2022 Jul 6;10:925402. doi: 10.3389/fpubh.2022.925402 (PMC9298888; doi:10.3389/fpubh.2022.925402)

## Supplementary Material 2

### Null and Preferential Models

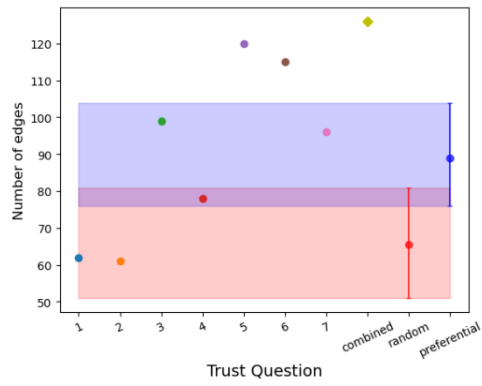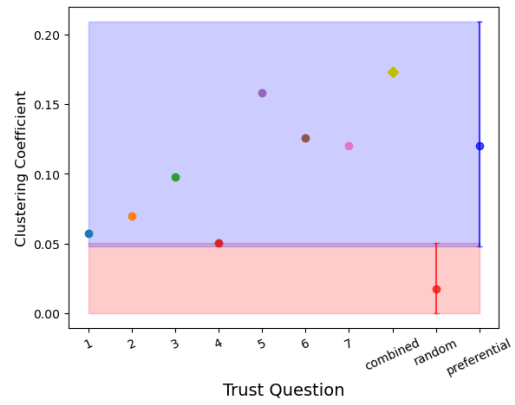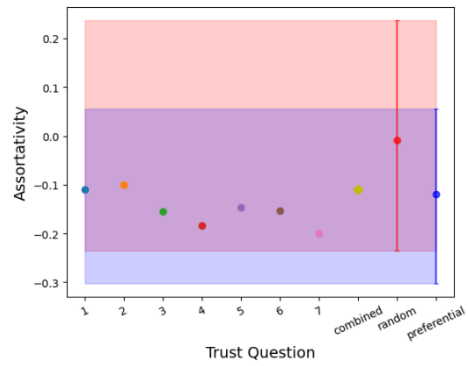

Supplement: Supplementary file 2 [file Data_Sheet_2.pdf]
